# Awareness of hypertension-related complications and its determinants among adult hypertensive patients in Ethiopia

**DOI:** 10.3389/fcvm.2025.1563249

**Published:** 2025-08-29

**Authors:** Habtam Reda Chekol, Negesu Gizaw Demissie, Chilot Kassa Mekonnen

**Affiliations:** Department of Medical Nursing, School of Nursing, College of Medicine and Health Sciences, University of Gondar, Gondar, Ethiopia

**Keywords:** awareness, complications, risk factors, blood pressure, hypertension, Gondar, Ethiopia

## Abstract

**Background:**

Hypertension is a critical medical condition that substantially raises the risk of developing heart, brain, kidney, and other organ-related diseases. Despite its significance, limited information is available regarding patients' awareness of hypertension complications and associated factors in the study area. Consequently, this study sought to evaluate the awareness levels of hypertensive patients concerning the risk factors and complications of hypertension.

**Objective:**

This study aimed to assess the awareness of hypertension-related complications and the factors influencing it among adult hypertensive patients.

**Methods:**

A cross-sectional institutional study was conducted among hypertensive patients. Bivariable and multivariable logistic regression analyses were employed, using odds ratios with 95% confidence intervals and *p*-values ≤ 0.05 to determine statistically significant associations.

**Results:**

The study included 422 participants, achieving a 100% response rate. Among the participants, only 42.4% (95% CI: 37.7–47.2) were knowledgeable about hypertension risk factors, complications, and prevention measures. The key predictors of awareness were being an urban resident [AOR = 7.20, 95% CI (3.77–13.76)], attaining a college or higher education level [AOR = 2.28, 95% CI (1.14–6.58)], working as a government employee [AOR = 2.97, 95% CI (1.66–7.05)], using social media [AOR = 6.01, 95% CI (3.37–10.71)], receiving advice from health professionals [AOR = 4.17, 95% CI (2.06–5.88)], attending all follow-up appointments [AOR = 1.88, 95% CI (1.04–2.47)], and having been diagnosed for ten or more years [AOR = 2.33, 95% CI (1.15–4.72)].

**Conclusion:**

The study highlighted a significant gap in awareness about hypertension risk factors and complications, with only two-fifths of participants demonstrating adequate knowledge. These findings underscore the need for targeted public health interventions, particularly focusing on rural communities, to bridge the awareness gap.

## Introduction

Hypertension is a serious medical condition that significantly increases the risks of heart, brain, kidney and other diseases. It is diagnosed when the systolic and diastolic blood pressure readings on two occasions are ≥140 mmHg by ≥90 mmHg (1). An estimated 1.28 billion adults aged 30–79 years worldwide suffer from hypertension, most (two-thirds) living in low- and middle-income countries (1). Hypertension is a principal cause of cardiovascular diseases (CVDs) such as myocardial infarction and stroke worldwide (2). The Meta-analysis outcomes from 9 studies in Ethiopia showed that the prevalence of hypertension among the Ethiopian population was 19.6% of which 23.7% was in rural and 14.7% was urban combined population while 20.6% was in males and 19.2% was in females (3). The excessive pressure on the artery walls caused by high blood pressure can damage blood vessels and body organs. The higher the blood pressure and the longer it goes uncontrolled, the greater the damage. Hypertension can lead to complications including Heart attack or stroke, Aneurysm, Heart failure, Kidney problems, Eye problems, Metabolic syndrome, Changes in memory or understanding, and Dementia (4).

The systematic review reveals low levels of awareness and treatment of hypertension and even lower levels of control. Custom-made research is required to discover the particular causes behind these low levels of awareness, treatment, and control and to notify the policy design for upgrading the consequences of hypertensive patients in Africa (5).

Hypertension remains a massive public health and economic burden globally regardless of recent improvements in the trend of blood pressure control. It is an independent prognosticator of cardiovascular disease and entirely causes mortality (6). The prevalence and the health significance of uncontrolled hypertension make it among the world's most deadly diseases (7). The studies have explored that hypertension accounts for 45% of all heart disease deaths and 51% of all stroke-related deaths, which together are the biggest causes of morbidity and mortality worldwide. Annually, there are 17 million deaths due to cardiovascular disease worldwide, of which 9.4 million are attributable to complications of raised BP (8). The systematic analysis shows that the prevalence of hypertension is increasing in Africa and many hypertensive individuals are not aware of their condition. The prevalence of hypertension in Africa was 19.7%, 27.4% and 30.8% in 1990, 2000 and 2010 year and the awareness rate of hypertension was 16.9%, 29.2% and 33.7%, respectively (9). Obtaining information about the level of awareness toward hypertension-related complications is the primary stage in formulating a preventive program for any health problem (10, 11).

A study in Karachi, south Asia reported that the level of awareness toward hypertension-related complications among hypertensive patients was, 100%, 95.5%, 59.1% and 54.5% for stroke, heart diseases, kidney disease and eye disease respectively (12). Moreover, the study in Rohilkhand region, India showed that the level of awareness toward hypertension-related complications among hypertensive patients was, 66.7%, 35.71%, 34.7% and 19% for heart damage, kidney damage, brain damage and others respectively (13). The study done in Dallas County, Texas and Pokhara, western Nepal reveals that the overall level of awareness toward hypertension-related complications among hypertensive patients was 64.4% and 73.1% respectively (14–16). Furthermore, a study done in Estonia, Russia showed that the level of awareness toward hypertension-related complications among hypertensive patients was 24.9%, 21.1% and 17.9% for stroke, cerebral infarction and cardiac infarction respectively (17).

A study done in Jaffna Peninsula, northern Sri Lanka brought that the level of awareness toward hypertension-related complications among hypertensive patients was 48.2% of which, 23.7%, 42.2%, 46.7% and 13.8% for kidney damage, heart damage, brain damage and eye damage respectively (18). The research conducted in southern Tanzania brought that the level of awareness toward hypertension-related complications among hypertensive patients was, 58.9%, 83.3%, 32.0%, 44.2% and 36.9% for Stroke, Heart diseases, Renal diseases, Eye disease and Arterial diseases respectively (19).

However, to the best of the researcher's knowledge, little study has been conducted that has addressed awareness of risk factors, hypertension-related complications and associated factors among adult hypertensive patients on follow-up in Ethiopia, particularly in this study area.

Therefore, this study aimed to determine awareness of hypertension-related complications, and associated factors among adult hypertensive patients during follow-up in the study area.

## Method and material

### Study area and period

The study was conducted at the University of Gondar Comprehensive Specialized Hospital. The milestone of medical care at the site of the current University of Gondar comprehensive specialized hospital was laid in 1920 when the Italian consulate South of Qusquam was established. University of Gondar Comprehensive Specialized Hospital is in the Central Gondar zone of Amhara National Regional State, Northwest Ethiopia. It is located 780 km from Addis Ababa (the capital city of Ethiopia) and 180 km from Bahir Dar (the capital city of the Amhara region). The hospital was established in 1954 as a Public Health College and Training Center. It is one of the regional referral and teaching hospitals that served more than 7 million people. The hospital had over 12 departments, 40 outpatient follow-up clinics, and 953 inpatient beds. At the University of Gondar comprehensive specialized hospital hypertension follow-up clinic, over 5,000 hypertensive patients received care, and over 1,550 patients received care every month (20). The study was conducted from April 15–May 15/2023.

### Study design

An institution-based cross-sectional study was conducted among hypertensive patients in a follow-up clinic at the University of Gondar Comprehensive Specialized Hospital, Gondar, Ethiopia, 2023.

### Source and study population

All adult hypertensive patients who came to the University of Gondar Comprehensive Specialized Hospital were the source population. Whereas, the study population was selected from adult hypertensive patients in a follow-up clinic at the University of Gondar Comprehensive Specialized Hospital during the study period.

### Inclusion and exclusion criteria

All hypertensive patients aged 18 years and above have been on follow-up at the University of Gondar Comprehensive Specialized Hospital. However, patients unable to communicate because of serious medical illness during the data collection period were excluded.

### Sample size determination

The sample size for the first objective of awareness about hypertension complications, risk factors and prevention measures was determined by using a single population proportion formula considering the following: 95% confidence interval (CI), 50% population proportion since no previous study and 5% marginal error.

n=(Zα/P2)2*p(1−p)/(d)2

*n* = the initial sample size, Z *α*/2 = Standardized normal distribution value for the 95% CI, =1.96, *P* = proportion (50%), d = margin of error 5% then substitute the numbers

The total sample size “n” was calculated as: n = (1.96 × 1.96) × 0.5 (1–0.5))/0.05 × 0.05 = 384, by adding a 10% non-response rate the final sample size was 422. The second objective of identifying factors associated with awareness was not attempted since there is no previous study.

### Sampling technique and procedures

A systematic random sampling method was used to select the study participants, taking into consideration the patient flow pattern at the hypertension clinic. On average, 1,558 patients received hypertension care each month at the University of Gondar Comprehensive Specialized Hospital. Then, to determine the sampling interval (k), the total population of 1,558 was divided by the desired sample size of 422, yielding approximately 3.7, which was rounded to 4. The first participant was randomly selected using a lottery method from numbers 1–4. Subsequently, every 4th patient was selected for participation. Each selected patient was first asked for their consent to take part in the study, and those who agreed were interviewed after completing their clinic visit.

### Operational definitions

**Awareness towards hypertension-related complications:** A total of 22 questions was used to assess patients' awareness of hypertension complications (5 questions), hypertension risk factors (11 questions), and prevention measures (6 questions). Each correct response to a question was worth one point, while each incorrect response was worth zero points. In this case, the minimum score was 0, whereas the maximum was 22. Then patients who responded to 0–15 correct questions were categorized as not aware, and those who responded to 16–22 correct answers, were aware (19).

**Regularity in follow-up visits**: A participant was considered irregular in treatment follow-up visits if he/she had missed more than three out of 10 scheduled/advised follow-up visits, otherwise regular (21).

### Data collection tools and procedures

The questionnaire was prepared after reviewing relevant literature on the problem under study and a structured questionnaire was used. It contains five sections one socio-demographic related, two health and clinical related, three about hypertension risk factors, four about hypertension complications, and five hypertension prevention measures. The Amharic language version of the questionnaire was used for data collection purposes. Three BSc Nurses who have experience in data collection were involved in the data collection. One MSc holder health professional was recruited as supervisor. Data was collected by using a pretested questionnaire through face-to-face interviews.

### Data quality control

The questionnaire was pretested on 5% (21 individuals) of the total sample size, selected from hypertensive patients attending the follow-up clinic at Felege Hiwot Referral Hospital. Following the pretest, adjustments were made to ensure the questionnaire's internal validity before its use in actual data collection. Data collectors and supervisors received one day of training on the study instrument and data collection procedures. Both the supervisor and the principal investigator oversaw the proper implementation of data collection and reviewed the completeness and logical consistency of the study tool daily. Additionally, the principal investigator carefully reviewed and cleaned the data during entry before commencing analysis. The questionnaire was translated into Amharic, the local language, and portions of it were distributed for the pretest.

### Data processing and analysis

The data was reviewed for consistency and completeness, coded, and entered into Epi Data version 4.6 by the principal investigator, and then exported to SPSS version 26 for analysis. Statistical summaries were used to describe all study variables. Simple descriptive statistics, such as mean and standard deviation, were applied for quantitative variables, while frequencies and percentage distributions were used for categorical variables. The relationship between dependent and independent variables was initially examined using bivariate analysis, with variables showing a *p*-value of less than 0.25 considered for multivariable logistic regression analysis. In the multivariable logistic regression, the strength of associations was interpreted using Adjusted Odds Ratios (AOR) with 95% confidence intervals (CI). Statistical significance was set at a 5% level (*p* < 0.05). The model's suitability was evaluated using the Hosmer-Lemeshow goodness-of-fit test, and multicollinearity among explanatory variables was assessed through collinearity diagnostics using the variance inflation factor.

## Result

### Socio-demographic and clinically related characteristics of participants

A total of 422 participants were enrolled in the study, with a 100% response rate. The mean and standard deviation (SD) age of the participants was 56.23 ± 16:62 years respectively. The majority of the respondents 284 (67.3%) were urban dolers. Regarding marital status, the majority of participants (67.5%) were married (Table 1).

**Table 1:** Socio-demographic and other characteristics of participants with hypertension attending UoGCSH, 2023 (*n* = 422).

Variables	Category	Frequency (*n*)	Percent (%)
Age group in years	20–29	35	8.3
	30–39	59	14.0
	40–49	50	11.8
	50–59	68	16.1
	60–69	109	25.8
	>=70	101	23.9
Sex	Male	154	36.5
	Female	268	63.5
Residence	Urban	284	67.3
	Rural	138	32.7
Marital status	Single	43	10.2
	Married	285	67.5
	Divorced	68	16.1
	Widowed	26	6.2
Educational status	Can't read & write	98	23.2
	Can read & write	80	19.0
	Primary school	57	13.5
	Secondary school	85	20.1
	College & above	102	24.2
Occupational status	Farmer	124	29.4
	Housewife	118	28.0
	Private employee	84	19.9
	Government employee	96	22.7
Health insurance	Yes	380	90.0
	No	42	10.0
Support for medical fee	Yes	278	65.9
	No	144	34.1
Use social media	Yes	136	32.2
	No	286	67.8

Among 422 participants, most of them (65.2%) have no family history of hypertension. Besides 61.4% of participants had no previous hypertension complication. In addition, 40.3% of participants missed their Follow-up in the previous year (Table 2).

**Table 2:** Clinical-related characteristics of participants with hypertension attending the UoGCSH, 2023 (*n* = 422**)**.

Variables	Category	Frequency (*n*)	Percent (%)
Family history of Hypertension	Absent	275	65.2
	Not sure	96	22.7
	Present	51	12.1
Previous hypertension complications	Yes	163	38.6
	No	259	61.4
Advised towards hypertension	Yes	278	65.9
	No	144	34.1
Follow-up missed in previous year	Yes	170	40.3
	No	252	59.7
Duration of hypertension since diagnosis	<=2 years	114	27.0
	3–4years	73	17.3
	5–9years	74	17.5
	>=10years	161	38.2

### Awareness of hypertension risk factors, complications, and prevention measures

The overall awareness in this was, only 42.4% (95% CI: 37.7, 47.2) of them were aware towards risk factors and complications. Furthermore, 214(50.71%) of the participants had an awareness of hypertension risk factors. Of these, the vast majority 357(84.6%) of the participants knew about stress as one of the risk factors of hypertension followed by 344(81.5%) that stated family history as a risk factor. Besides, 189(44.79%) of the participants had awareness about hypertension complications with 201(47.6%) of the participants stating stroke as a complication. Moreover, 292(69.19%) of the participants had an awareness of hypertension prevention measures. The participants reported that taking regular medication is one of the prevention measures (Table 3).

**Table 3:** Distribution of awareness about hypertension risk factors, complications, and prevention measures among hypertensive patients.

Question	Yes (%)	No (%)
Sex is a risk factor for hypertension	61 (14.5)	361 (85)
Age is a risk factor for hypertension	164 (38.9)	258 (61.1)
Race is a risk factor for hypertension	56 (13.3)	366 (86.7)
Family history is a risk factor for hypertension	344 (81.5)	78 (15.5)
Uncontrolled diabetes mellitus is a risk factor for hypertension	203 (48.1)	219 (51.9)
Excessive salt intake is a risk factor for hypertension	340 (80.0)	82 (19.4)
Excessive alcohol intake is a risk factor for hypertension	308 (73.0)	114 (27.0)
Physical inactivity is a risk factor for hypertension	264 (62.6)	158 (37.4)
Obesity is a risk factor for hypertension	232 (55.0)	190 (45.0)
Smoking is a risk factor for hypertension	285 (67.5)	137 (32.5)
Stress is a risk factor for hypertension	357 (84.6)	65 (15.4)
Overall awareness about the risk of hypertension	214 (50.71)	208 (49.29)
Awareness of complications	Yes (%)	No (%)
Hypertension could cause stroke/brain damage	201 (47.6)	221 (52.4)
Hypertension will lead you to heart disease	194 (46.0)	228 (54.0)
Hypertension could lead to eye disease	191 (45.3)	231 (54.7)
Hypertension could lead to kidney disease	165 (39.1)	257 (60.9)
Hypertension could lead to Arterial disease	195 (46.2)	227 (53.8)
Overall awareness about complications	189 (44.79)	233 (55.21)
Awareness towards hypertension prevention	Yes (%)	No (%)
Reduction in salt intake	251 (59.5)	171 (40.5)
Stopping/Reducing alcohol intake	272 (64.5)	150 (35.5)
Doing exercise	275 (65.2)	147 (34.8)
Weight reduction	279 (66.1)	143 (33.9)
Cessation of smoking	285 (67.5)	137 (32.5)
Taking regular medication	392 (92.9)	30 (7.1)
Overall awareness of prevention measures	292 (69.19)	130 (30.81)

### Association between independent variables with awareness of hypertension-related complications

The relationship between awareness and the independent variables was evaluated using the Chi-square test. As Table 4 indicates there was a significant association (*p*-value ≤ 0.05) between awareness and factors such as residence, educational level, occupation, receiving advice from healthcare professionals, adherence to follow-up appointments, exposure to social media, and the duration of hypertension since diagnosis.

**Table 4:** Tests of association of independent variables with awareness of hypertension complications.

Variables	Awareness		Total (%)
	Not aware (%)	Aware (%)	
Prevalence	57.6[95% CI(52.8,61.9)]	42.4[95% CI(38.1,47.2)]	100
Age group in years			
20–29	18 (51.4)	37 (48.6)	100
30–39	22 (37.3)	37 (62.7)	100
40–49	23 (46.0)	27 (54.0)	100
50–59	40 (58.8)	28 (41.2)	100
60–69	70 (64.2)	39 (35.8)	100
≥70	70 (69.3)	31 (30.7)	100
	* X²* = 20.93, df = 5, *p* = 0.001		
Sex			
Male	93 (60.4)	61 (39.6)	100
Female	150 (56.0)	118 (44.0)	100
	*X²* = 0.78, df = 1, *p* = 0.38		
Residence			
Urban	127 (44.7)	157 (55.3)	100
Rural	116 (84.1)	22 (15.9)	100
	*X²* = 58.85, df = 1, *p* = 0.001		
Marital status			
Single	19 (44.2)	24 (55.8)	100
Married	174 (61.1)	111 (38.9)	100
Divorced	34 (50.0)	34 (50.0)	100
Widowed	16 (61.5)	10 (38.5)	100
	*X²* = 6.33, df = 3, *p* = 0.09		
Educational status			
Can't read and write	65 (66.3)	33 (33.7)	100
Can read and write	45 (56.3)	35 (43.8)	100
Primary school	42 (73.7)	15 (26.3)	100
Secondary school	51 (60.0)	34 (40.0)	100
College and above	40 (39.2)	62 (60.8)	100
	*X²* = 23.47, df = 4, *p* < 0.001		
Occupational status			
Farmer	87 (70.2)	37 (29.8)	100
Housewife	72 (61.0)	46 (39.0)	100
Private employee	47 (56.0)	37 (44.0)	100
Government employee	37 (38.5)	59 (61.5)	100
	*X²* = 22.94, df = 3, *p* < 0.001		
Health insurance			
Yes	223 (58.7)	157 (41.3)	100
No	20 (47.6)	22 (12.3)	100
	*X²* = 1.89, df = 1, *p* = 0.169		
Advice from HCPs			
Yes	147 (52.9)	131 (47.1)	100
No	96 (66.7)	48 (33.3)	100
	*X²* = 7.39, df = 1, *p* < 0.007		
Social support			
Yes	162 (58.3)	116 (41.7)	100
No	81 (56.3)	63 (43.7)	100
	*X²* = 0.159, df = 1, *p* = 0.690		
Follow-up missed			
Yes	110 (64.7)	60 (35.3)	100
No	133 (52.8)	119 (47.1)	100
	*X²* = 5.914, df = 1, *p* < 0.015		
Social media exposure			
Yes	37 (27.2)	99 (72.8)	100
No	206 (72.0)	80 (28.0)	100
	*X²* = 75.813, df = 1, *p* < 0.001		
Duration of hypertension since diagnosis			
≤2 years	71 (62.3)	43 (37.7)	100
3–4 years	41 (56.2)	32 (43.8)	100
5–9 years	50 (67.6)	24 (32.4)	100
≥10 years	81 (50.3)	80 (49.7)	100
	*X²* = 7.597, df = 3, *p* = 0.025		

Moreover, the association between independent variables and awareness of hypertension-related complications revealed that awareness decreased with age, starting at 48.6% in the 20–29 age group and dropping to 30.7% in those aged ≥70 years (*p* < 0.001). Urban residents' demonstrated higher awareness compared to rural residents (*p* ≤ 0.001). Females had greater awareness than males, though the difference was not statistically significant (*p* = 0.38). Marital status showed a decline in awareness from 55.8% among single individuals to 38.5% among widowed individuals (*p* = 0.09). The highest awareness, at 60.0%, was observed among individuals with the highest educational attainment (*p* ≤ 0.001). Similarly, those exposed to media reported significantly greater awareness (72.8%) compared to those without media exposure (*p* ≤ 0.001), as shown in Table 4.

### Factors associated with awareness of respondents towards hypertension-related complications

In the Bivariable analysis, variables with a *p*-value of less than 0.25, such as age, residence, marital status, educational level, occupational status, social media exposure, missed follow-ups, health insurance, advice on hypertension, and duration of hypertension since diagnosis, were identified as candidates for multivariable logistic regression.

The multivariable analysis revealed that age group, residence, educational level, occupational status, social media exposure, adherence to follow-ups, and the duration of hypertension since diagnosis were significant factors associated with awareness. Specifically, individuals aged 60–69 had reduced odds of awareness, with adjusted odds ratios of 0.35 [AOR = 0.35, 95% CI (0.13, 0.91)] and 0.37 [AOR = 0.37, 95% CI (0.14, 0.98)] for awareness about hypertension-related complications. Urban residents were 7.02 times more likely to be aware of hypertension-related complications, risk factors, and prevention measures compared to rural residents [AOR = 7.02, 95% CI (3.77, 13.76)].

Educational status was also significant; individuals with literacy skills had lower odds of awareness [AOR = 0.22, 95% CI (0.11, 0.43)], as did those with primary school education [AOR = 0.36, 95% CI (0.17, 0.74)], while those with college-level education or higher were 2.28 times more likely to be aware [AOR = 2.28, 95% CI (1.14, 6.58)]. Government employees were 2.97 times more likely to have awareness compared to farmers [AOR = 2.97, 95% CI (1.66, 7.05)].

Social media usage significantly increased awareness odds by 6.01 times compared to non-users [AOR = 6.01, 95% CI (3.37, 10.71)]. Likewise, hypertensive patients who received professional advice had 4.17 times higher odds of awareness [AOR = 4.17, 95% CI (2.06, 5.88)] compared to those who did not. Adherence to follow-ups increased awareness by 1.88 times [AOR = 1.88, 95% CI (1.04, 2.47)]. Lastly, patients diagnosed with hypertension for ten years or more were 2.28 times more likely to be aware compared to those diagnosed for two years or less [AOR = 2.28, 95% CI (1.19, 4.33)] (Table 5).

**Table 5:** Bivariable and multivariable logistic regression analysis to assess predictors of awareness toward hypertension risk factors and complications among hypertensive patients at the UOGCSH, 2023 (*n* = 422).

Variables	Category	Awareness		COR (95% CI)	AOR (95% CI)
		Not Aware (%)	Aware (%)		
Age in years	20–29	18 (51.4)	37 (48.6)	1	1
	30–39	22 (37.3)	37 (62.7)	1.78 (0.76, 4.16)	1.41 (0.49, 4.09)
	40–49	23 (46.0)	27 (54.0)	1.24 (0.52, 2.95)	1.23 (0.43, 3.54)
	50–59	40 (58.8)	28 (41.2)	0.74 (0.33, 1.68)	0.94 (0.34, 2.60)
	60–69	70 (64.2)	39 (35.8)	0.59 (0.27, 1.27)	0.35 (0.13, 0.91)*
	>=70	70 (69.3)	31 (30.7)	0.47 (0.21, 1.03)	0.37 (0.14, 0.98)*
Residence	Urban	127 (44.7)	157 (55.3)	6.52 (3.91, 10.88)	7.20 (3.77, 13.76)*
	Rural	116 (84.1)	22 (15.9)	1	1
Marital status	Single	19 (44.2)	24 (55.8)	1	1
	Married	174 (61.1)	111 (38.9)	0.51 (0.26, 0.97)	0.56 (0.34, 2.45)
	Divorced	34 (50.0)	34 (50.0)	0.79 (0.37, 1.71)	0.62 (0.29, 1.57)
	Widowed	16 (61.5)	10 (38.5)	0.50 (0.18, 1.34)	0.38 (0.16, 2.67)
Educational status	Can't read & write	65 (66.3)	33 (33.7)	1	1
	Can read & write	45 (56.3)	35 (43.8)	1.53 (0.83, 2.82)	0.22 (0.11, 0.43)*
	Primary school	42 (73.7)	15 (26.3)	0.70 (0.34, 1.45)	0.36 (0.17, 0.74)
	Secondary school	51 (60.0)	34 (40.0)	1.31 (0.72, 2.40)	1.15 (0.26, 3.35)
	College & above	40 (39.2)	62 (60.8)	3.05 (1.71, 5.44)	2.28 (1.14, 6.58)*
Occupational status	Farmer	87 (70.2)	37 (29.8)	1	1
	Housewife	72 (61.0)	46 (39.0)	1.50 (0.88, 2.56)	1.47 (0.82, 2.89)
	Private employee	47 (56.0)	37 (44.0)	1.85 (1.04, 3.30)	1.35 (0.63, 3.07)
	Government employee	37 (38.5)	59 (61.5)	3.75 (2.14, 6.58)	2.97 (1.66, 7.05)*
Health insurance	Yes	223 (58.7)	157 (41.3)	0.64 (0.34, 1.21)	0.32 (0.12, 2.99)
	No	20 (47.6)	22 (12.3)	1	1
Advice from HCPs	Yes	147 (52.9)	131 (47.1)	1.78 (1,17, 2.71)	4.17 (2.06, 5.88)*
	No	96 (66.7)	48 (33.3)	1	1
Social support	Yes	162 (58.3)	116 (41.7)	0.92 (0.61, 1.38)	
	No	81 (56.3)	63 (43.7)	1	1
Follow-up missed	Yes	110 (64.7)	60 (35.3)	1	1
	No	133 (52.8)	119 (47.1)	1.64 (1.10, 2.45)	1.88 (1.04, 2.47)*
Social media exposure	Yes	37 (27.2)	99 (72.8)	6.89 (4.36, 10.89)	6.01 (3.37, 10.71))*
	No	206 (72.0)	80 (28.0)	1	1
Duration of hypertension since diagnosis	<=2 years	71 (62.3)	43 (37.7)	1	1
	3–4years	41 (56.2)	32 (43.8)	1.29 (0.71, 2.34)	0.69 (0.32, 1,51)
	5–9years	50 (67.6)	24 (32.4)	0.79 (0.43, 1.47)	0.59 (0.25, 1.44)
	>=10years	81 (50.3)	80 (49.7)	1.63 (1.00, 2.66)	2.28 (1.19, 4.33)*

COR, crude odds ratio; AOR, adjusted odds ratio.

*Significantly associated with the outcome variable.

## Discussion

This study found that most hypertensive patients have low awareness towards risk factors and complications of hypertension. In general, only 42.4% (95% CI: 37.7, 47.2) of them had good knowledge towards stroke risk factors. This finding is higher than the study conducted in Tanzania (19). This might be due to the reason that the number of participants who were in the higher education level in our study is much higher than in the previous study, and it may be because the education level has a significant impact on awareness. Besides, participants who had been living with the disease for 10 years or more in the current study were higher, while in the previous study were less. This may create a difference in awareness. In other words, five years have passed since that previous study was conducted, and time itself makes a difference. In another way, this finding is lower than the study conducted in India (13). This may be because the country where the previous study was conducted had better access to medical care and the number of people who participated in the study was small compared with the current study. A study in Iran revealed that 80.7% of hypertensive patients were aware of hypertension risk factors and complications if left uncontrolled (22). The other study in China (23) revealed that 47.9% of the participants were aware of hypertension risk factors and related complications.

In multivariable logistic regression analysis, the variables age group, residence, educational level, occupational status, social media exposure, adherence to follow-ups, and the duration of hypertension since diagnosis were significant factors associated with awareness. Participants aged 60–69 years were 65% less likely to be aware of hypertension-related complications, with an adjusted odds ratio of 0.35. Similarly, those aged 70 years and older were 63% less likely to have such awareness, with an adjusted odds ratio of 0.37. A possible justification for the lower awareness of hypertension-related complications among older age groups could stem from a combination of factors, including cognitive decline, limited access to information, social isolation, and a focus on managing existing health conditions (24). For instance, as age increases, age-related cognitive decline might hinder their ability to process, retain, and recall information about health conditions, and reduce awareness levels (25). Moreover, older individuals often have multiple health conditions and might focus more on managing immediate health issues rather than seeking preventive or educational information about other potential complications.

Urban residents demonstrated significantly greater awareness, being 7.20 times more likely to understand hypertension-related complications, risk factors, and preventive measures compared to their rural counterparts. This might be because people living in urban areas had easy access and more health-seeking behaviour than people in rural areas. The markedly higher awareness of hypertension-related complications, risk factors, and preventive measures among urban residents compared to their rural counterparts can be explained by several factors, including improved access to healthcare facilities, increased exposure to health education campaigns, easier access to information, and the presence of professional health networks (26).

Awareness was also influenced by educational attainment in which individuals with literacy skills had 78% lower odds of awareness while those with primary education had 64% lower odds. In contrast, individuals with college-level education or higher had 2.28 times greater odds of being aware. This is because individuals with lower educational attainment may have had fewer opportunities to engage in health education programs or access reliable health information, which often requires higher levels of comprehension (27). It would also be because persons with higher levels of education are more likely to seek medical attention, comprehend written and visual instructions, and educate themselves about disease. This was supported by a previous study conducted in Tanzania (19).

Using social media boosted awareness odds by 6.01 times compared to non-users. This was possible because those who use social media have health literacy and increased awareness about hypertension risk factors, complications, and preventive measures (28). This might be due to the reason that people who use social media are more aware of hypertension because they read the World Health Organization or National Ministry of Health guidelines that are released on various social media platforms regularly (29). Government employees were 2.97 times more likely to have awareness compared to farmers. The possible reasons for this discrepancy might be government employees typically require formal education to qualify for their roles, which may make them more likely to engage with information and have greater cognitive awareness of relevant topics. However, farmers, especially in rural or less developed areas, may have lower levels of formal education, limiting their access to information about the potential complications of hypertension (30).

Similarly, hypertensive patients who received professional advice had 4.17 times greater awareness. Advice from healthcare professionals is often perceived as authoritative and reliable, leading patients to take the information more seriously. Professionals can tailor advice to the patient's specific condition, ensuring the information is understandable and directly applicable (31, 32). In other way, participants who did not miss their follow-ups while adhering to follow-ups increased awareness by 1.88 times to care and were more aware as compared to those who missed their follow-ups. Regular follow-ups foster a structured routine and sense of responsibility, enhancing patients' understanding of care requirements and promoting proactive health management. They provide opportunities for engagement with healthcare providers, who reinforce key aspects of care like medication adherence, lifestyle changes, and symptom monitoring. Patients who miss their follow-ups lose opportunities for education and reinforcement, reducing awareness and understanding of potential complications of hypertension (33, 34).

Finally, patients diagnosed with hypertension for 10 years or more were 2.28 times more likely to be aware compared to those diagnosed for two years or less. Hypertension patients who have more than or equal to ten years duration since diagnosis were more aware as compared with hypertension patients who have less than or equal to two years duration. This might be because, people who have lived with the disease for a long time frequently discuss it with their doctors or other care providers, and they have a better understanding since they have experienced the problem or have witnessed a friend who has experienced it (33).

### Clinical implication of the study

This study highlights the importance of proactive strategies to improve awareness of hypertension, its risks, and complications among patients. Healthcare providers can organize targeted campaigns, including educational sessions, social media outreach, and informative materials like posters, to promote blood pressure control and healthy lifestyle choices. These initiatives empower patients to manage their health effectively, benefiting both individuals and the broader community by reducing hypertension-related complications and easing the healthcare system's burden.

## Conclusion

The study highlighted a substantial gap in awareness concerning the risk factors and complications associated with hypertension. Only approximately two-fifths of the participants demonstrated an adequate level of knowledge in these areas, indicating that a majority lacked essential information about the condition. This lack of awareness can have serious implications for early detection, treatment adherence, and overall disease management.

The findings emphasize the urgent need for targeted public health interventions aimed at improving health literacy, especially in underserved and rural communities where access to healthcare information may be limited. Strengthening education campaigns, incorporating culturally appropriate materials, and engaging community health workers could be effective strategies to bridge this awareness gap and ultimately reduce the burden of hypertension-related complications. Key factors influencing awareness included urban residence, higher education levels, employment as a government worker, access to social media, guidance from healthcare professionals, adherence to follow-up appointments, and having been diagnosed with hypertension for 10 years or more.

## Data Availability

The original contributions presented in the study are included in the article/Supplementary Material, further inquiries can be directed to the corresponding author.
